# Complementary proteomic approaches reveal mitochondrial dysfunction, immune and inflammatory dysregulation in a mouse model of Gulf War Illness

**DOI:** 10.1002/prca.201600190

**Published:** 2017-05-12

**Authors:** Zuchra Zakirova, Jon Reed, Gogce Crynen, Lauren Horne, Samira Hassan, Venkatarajan Mathura, Michael Mullan, Fiona Crawford, Ghania Ait‐Ghezala

**Affiliations:** ^1^ Department of Genomics The Roskamp Institute Sarasota FL USA; ^2^ Icahn School of Medicine at Mount Sinai New York NY USA; ^3^ Boehringer Ingelheim Pharmaceuticals, Inc. Ridgefield CT USA; ^4^ University of Central Florida College of Medicine Orlando FL USA; ^5^ James A. Haley Veterans Hospital Tampa FL USA

**Keywords:** Gulf War, iTRAQ, mitochondrial dysfunction, SIDL, MS/MS

## Abstract

**Purpose:**

Long‐term consequences of combined pyridostigmine bromide (PB) and permethrin (PER) exposure in C57BL6/J mice using a well‐characterized mouse model of exposure to these Gulf War (GW) agents were explored at the protein level.

**Experimental design:**

We used orthogonal proteomic approaches to identify pathways that are chronically impacted in the mouse CNS due to semiacute GW agent exposure early in life. These analyses were performed on soluble and membrane‐bound protein fractions from brain samples using two orthogonal isotopic labeling LC‐MS/MS proteomic approaches—stable isotope dimethyl labeling and iTRAQ.

**Results:**

The use of these approaches allowed for greater coverage of proteins than was possible by either one alone and revealed both distinct and overlapping datasets. This combined analysis identified changes in several mitochondrial, as well as immune and inflammatory pathways after GW agent exposure.

**Conclusions and clinical relevance:**

The work discussed here provides insight into GW agent exposure dependent mechanisms that adversely affect mitochondrial function and immune and inflammatory regulation. Collectively, our work identified key pathways which were chronically impacted in the mouse CNS following acute GW agent exposure, this may lead to the identification of potential targets for therapeutic intervention in the future.

Long‐term consequences of combined PB and PER exposure in C57BL6/J mice using a well‐characterized mouse model of exposure to these GW agents were explored at the protein level. Expanding on earlier work, we used orthogonal proteomic approaches to identify pathways that are chronically impacted in the mouse CNS due to semiacute GW agent exposure early in life. These analyses were performed on soluble and membrane‐bound protein fractions from brain samples using two orthogonal isotopic labeling LC‐MS/MS proteomic approaches—stable isotope dimethyl labeling and iTRAQ. The use of these approaches allowed for greater coverage of proteins than was possible by either one alone and revealed both distinct and overlapping datasets. This combined analysis identified changes in several mitochondrial, as well as immune and inflammatory pathways after GW agent exposure. The work discussed here provides insight into GW agent exposure dependent mechanisms that adversely affect mitochondrial function and immune and inflammatory regulation at 5 months postexposure to PB + PER.

**Abbreviations**
BCA: Bicinchoninic acid (BCA)
COX: cytochrome c oxidase
COX6C: cytochrome c oxidase subunit 6C
DDA: data‐dependent acquisition
FDR: false discovery rate
GW: Gulf War
GWI: Gulf War Illness
IL‐1β: interleukin 1β
IL‐10: interleukin 10
IL‐17: interleukin 17
IPA: Ingenuity Pathway Analysis
MD: mitochondrial dysfunction
mTOR: mechanistic target of rapamycin
NDUF S4/B8: ubiquinone oxidoreductase subunit S4/B8
OXPHOS: oxidative phosphorylation
PB: pyridostigmine bromide
PER: permethrin
PI3K: phosphoinositide 3‐kinase
SDH A/B: succinate dehydrogenase complex flavoprotein subunit A/B
SIDL: stable isotope dimethyl labeling
TEAB: triethylammonium bicarbonate
TNF‐α: tumor necrosis family‐α

## 1 Introduction

Gulf War Illness (GWI) is a complex and multifaceted illness with CNS components such as memory deficits, neurological, and musculoskeletal problems. Gulf War (GW) agents, such as pyridostigmine bromide (PB) and pesticides such as permethrin (PER), have been implicated as key contributors to the etiology of GWI postdeployment to the Persian GW 1, 2, 3, 4. There are no effective treatments for GWI, and thus identification of biological pathways associated with the biological sequelae of GW agent exposure is vital to understanding the pathogenic mechanisms of GWI and for developing novel therapies for treatment.

**Clinical Relevance**

Two separate isotopic labeling LC‐MS/MS proteomic approaches, stable isotope dimethyl labeling and iTRAQ, were used to elucidate the biochemical changes that occurred in soluble and membrane‐bound protein fractions from brain samples of Gulf War agent exposed mice. Second, this work was carried out in order to identify pathways that are chronically impacted in the mouse CNS following acute Gulf War agent exposure, which may lead to the identification of potential targets for therapeutic intervention in the future.

Expanding on earlier work described in Zakirova et al. 4, in which we characterized neurobehavioral and neuropathological outcomes at 5 months postexposure, we have now used complementary orthogonal proteomic approaches to further characterize this model and identify pathways that are impacted in the CNS at this time point. These analyses were performed on soluble and membrane‐bound protein fractions from brain samples using two separate isotopic labeling LC‐MS/MS proteomic approaches—stable isotope dimethyl labeling (SIDL) and iTRAQ. The use of both approaches allowed for greater coverage of proteins than was possible by either one alone and revealed both distinct and overlapping datasets. The LC‐MS data were analyzed separately, uploaded separately to Ingenuity Pathway Analysis (IPA), and then a comparison analysis was performed using IPA software in order to assign biological significance to the protein changes that were identified. This comparison analysis of SIDL and iTRAQ identified changes in several mitochondrial, as well as immune and inflammatory, pathways after GW agent exposure at the long‐term time point. The work discussed here provides insight into GW agent exposure dependent mechanisms that adversely affect mitochondrial function and immune and inflammatory regulation at 5 months postexposure to PB + PER, and makes a case for incorporating complementary isotopic labeling and LC‐MS approaches into a proteomic study.

## 2 Materials and methods

### 2.1 Animals

All animal experiments were approved by the Roskamp Institute's Institutional Animal Care and Use Committee and conducted in accordance with the Office of Laboratory Animal Welfare and the Association for the Assessment and Accreditation of Laboratory Animal Care. Mice were purchased from Jackson Laboratories (Bar Harbor, Maine) and each mouse was individually housed in a controlled environment (regulated 14‐h day/10‐h night cycle) and maintained on a standard diet.

#### 2.1.1 Animal exposure

Twenty‐eight male C57BL6/J mice (12 weeks of age) were co‐administered either a 50 μL total volume of GW agents to a final dose of 0.7 mg/kg of PB (FisherSci) and 200 mg/kg of PER (25:75; Sigma Aldrich) in 100% DMSO (exposed mice; *n* = 14), or a 50 μL volume of vehicle (100% DMSO; control mice; *n* = 14) via intraperitoneal injection daily, for 10 days. These doses have been used in previous mouse studies showing adverse behavioral or pathological outcomes 3, 4, 5. All mice were euthanized at 5 months postexposure by blood withdrawal using a cardiac puncture, followed by a transcardial perfusion for approximately 3 min with PBS pH 7.4. Whole blood was collected in 1.5 mL Eppendorf tubes with 5 mM EDTA and centrifuged for 5 min at ≈1000 × *g* RCF. The brains were dissected and the hemispheres were separated on the midsagittal plane. The right hemisphere was snap frozen in liquid nitrogen and later processed for proteomics and biochemical analyses.

### 2.2 Protein extraction

All protein extracts and fractions were made from individual animals, and no pooling was used. Biological replicates were used at all discovery phase (i.e. LC‐MS/MS) and validation (i.e. immunoblot analyses) experiments.

**Figure 1:**
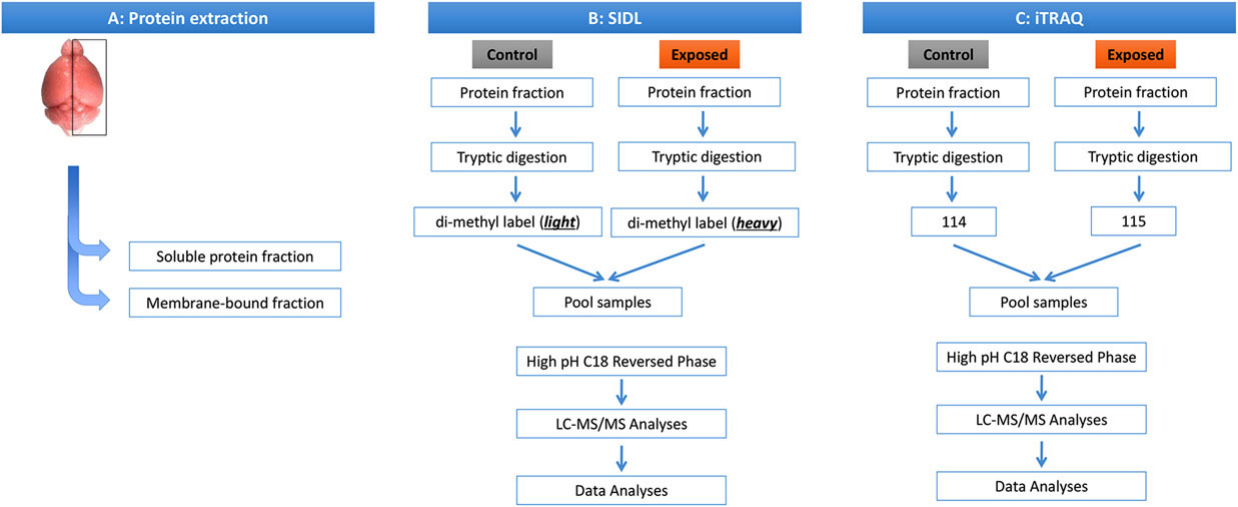
Proteomics workflow. Workflow (A) depicting protein extraction, and schematic of two proteomics experiments conducted using brain homogenates from PB + PER exposed mice and controls using (B) SIDL and (C) iTRAQ methodologies.

Individual right brain hemispheres from C57BL6/J animals exposed to either GW agents (PB + PER in DMSO) or vehicle (DMSO) were homogenized by sonication in PBS supplemented with a Protease/Phosphatase Inhibitor Cocktail (Thermo). The homogenates (*n* = 6/group) were clarified by centrifugation at 150 000 × *g* RCF for 30 min at 4°C. The supernatant (herein referred to as the “soluble” fraction) was transferred to a new tube and aliquoted for storage at −80°C until further use. The pellet (“membrane” fraction) was re‐suspended to a slurry via bath sonication in chilled 20 mM triethylammonium bicarbonate (TEAB; pH 8.0) supplemented with a Protease/Phosphatase Inhibitor Cocktail as above (proteomic work flow depicted in Fig. 1). PBS was selected as an initial extraction buffer over other commonly used proteomic extraction buffers which typically contain strong denaturants and/or high detergent concentrations, as it allows the extracts to be used for enzyme assays, and is compatible with other downstream “omic” approaches such as lipidomics, whereas the denaturing/detergent‐rich buffers are not. Protein concentrations were determined using the bicinchoninic acid (BCA) assay, and the concentrations/sample integrity was verified by SDS‐PAGE and Sypro Ruby staining.

#### 2.2.1 Tryptic digestion and isotopic labeling

Equal amounts (100 μg) of each sample (*n* = 6 per group (control/exposed)) from each fraction were spiked with an internal control (1 μg purified large subunit Spinach Rubisco), de‐salted using acetone precipitation, and the pellets re‐suspended in 10 μL of 25 mM TEAB and 1% w/v sodium deoxycholate. Individual samples (*n* = 6/group) were reduced and alkylated in 2 mM of tris (2‐carboxyethyl) phosphine, followed by addition of iodoacetamide to a final concentration of 25 mM. Each step was incubated in darkness for 30 min at 37°C. Sample volumes were adjusted to 100 μL by addition of 25 mM TEAB and 0.5 μg tosyl phenylalanyl chloromethyl ketone modified trypsin (1:200 enzyme‐to‐substrate ratio; Promega), and the samples were digested overnight at 37°C. A 5 μL process aliquot of the digested sample was evaluated by SDS‐PAGE and staining with Sypro Ruby to ensure the consistency and completeness of the digestion. Afterwards, 25 μg amounts of each individual tryptic digest were transferred to new tubes and labeled with iTRAQ reagents according to manufacturer's instruction (Applied Biosystems; see Fig. 1 for duplex labeling scheme). Corresponding 25 μg aliquots of the each digest were taken to new tubes and dried, followed by SIDL labeling according to Boersema et al. 6. Following their respective isotopic labeling schemes, the reactions were quenched, and the respective poolings were performed (Fig. 1). Sodium deoxycholate was depleted from the duplexed iTRAQ and SIDL pairs according to Masuda et al. 7, and the samples were taken to dryness in a vacuum centrifuge.

#### 2.2.2 2D LC‐MS/MS of iTRAQ‐ and SIDL‐labeled samples

To decrease the sample complexity at the peptide level, offline 2D high pH/low pH reversed liquid chromatography was performed using a method adapted from Gilar et al. 8. Briefly, dried samples were re‐suspended in 20 mM ammonium formate, pH 10, and loaded onto a C18 reversed phase spin column (50%) in binding buffer (20 mM ammonium formate, pH 10). The fractions were taken to dryness, and re‐suspended in LC‐MS mobile phase A (0.1% formic acid in water), and further de‐salted using C18 reversed phase ZipTips (Millipore). These were again taken to dryness in a vacuum centrifuge, and reconstituted in 25 μL of mobile phase A.

A Thermo Easy UPLC coupled to a Q‐Exactive Orbitrap (Thermo) was used for all proteomic experiments under the control of Xcalibur v 2.4 software in data‐dependent acquisition (DDA) mode.

For SIDL‐labeled samples, the samples were loaded onto a 0.1 × 30 mm PepMap C18 trapping column, and resolved using a 0.075 × 150 mm C18 (1.3 μm) PepMap column (Dionex) upon valve switching. Separation was performed over a 90‐min linear gradient from 2 to 45% mobile phase B (ACN) at 300 nL/min, and the column temperature was held to 35°C using a Phoenix S and T Butterfly Heater. The MS was run in DDA mode, using a Top 5 high/low strategy (full‐scan MS at 70 000 (full width at half maximum) resolution and 17 500 resolution for higher energy C‐trap dissociation MS^2^ spectra), and dynamic exclusion (60 s). This approach was the most suitable compromise between sensitivity (defined as the number of peptides identified) and quantitative accuracy (which relies on statistically reliable numbers of full‐scan spectra for peak integration).

For iTRAQ‐labeled peptides, both chromatographic and mass spectrometric methods were adjusted to accommodate the different technical demands of MS^2^‐based quantitation inherent to iTRAQ‐labeled samples. Following desalting on the trapping column, the iTRAQ‐labeled peptides were separated on a 0.075 × 500 mm C18 column (2 μm particle size; PepMap, Thermo) that was held at 40°C. The peptides were separated over a 4.5‐h linear gradient of increasing ACN, from 2 to 30% mobile phase B at 250 nL/min. DDA settings for the iTRAQ experiments were as follows: full‐scan MS resolution = 140 000 full width at half maximum at 200 *m/z*, full‐scan range = 380–1250 *m/z*, isolation width = 1.2 *m/z*, higher energy C‐trap dissociation relative collision energy = 29, a minimum *m/z* setting of 100 *m/z* was used for all MS^2^ spectra, MS^2^ resolution = 17 500, dynamic exclusion = 180 s, and a Top 15 high/low duty cycle was used for precursor ion selection. These settings, essentially the narrow isolation window and the ultra‐long gradient, were used to minimize the deleterious effects on quantitative accuracy that result from co‐isolation of isobaric precursors without resorting to MS^3^‐based methods.

### 2.3 LC‐MS data analysis

PMi Preview software (Protein Metrics) was used to survey the data files and, if necessary, to add other modifications to the search criteria. Preview results were also used to choose ideal precursor and fragment ion mass tolerances (4 ppm, 0.02 Da, respectively) as well as dynamic modifications. The Mouse FASTA protein database used in this study was downloaded from the Uniprot website on December 12, 2013. MS data were then searched via the SEQUEST and Byonic (Protein Metrics) search algorithms in Proteome Discoverer 1.4 (Thermo) using the following criteria for SIDL data: maximum allowed missed cleavages for trypsin = 2; static modifications: dimethyl residues = +28.031 or +36.076 Da (N‐terminus and K) and carbamidomethyl +57.021 Da (C); and dynamic modifications: oxidation +15.995 Da (M), methylation +14.016 Da of (E), carbamidomethyl/+57.021 Da (any N‐terminus and H).

For duplex iTRAQ data, the following settings were used: static modifications of iTRAQ‐2‐plex +144.102 (N‐terminus, K) and carbamidomethyl +57.021 (C); dynamic modifications were as follows: oxidation/+15.995 Da (M), methyl/+14.016 Da (E), deamidated/+0.984 Da (N, Q). Signal intensity cutoff for all experiments was zero, to avoid losing biologically important proteins that are unique to one condition and not the other. The Percolator feature of Proteome Discoverer was used to set a false discovery rate (FDR) of 0.01 for the SEQUEST searches; reverse database search was used to set FDR to 0.01 in the Byonic searches. Co‐isolating peptides were excluded and only unique peptides were used in quantification. All peptides used in further analysis were quantified in at least half of the runs (iTRAQ or SIDL). Peptides passing these cut off values were exported to JMP (SAS) 8.0.2 for data processing and statistical analysis. iTRAQ and SIDL datasets and result files are uploaded to PRIDE database (iTRAQ PXD005185, SIDL PXD005198).

#### 2.3.1 Statistical analyses of proteomic data

There were six biological replicates per treatment group, however there were no technical replicates for each MS run. The same biological replicates were used for both iTRAQ and SIDL experiments. For each peptide, *ln* transformed (PB + PER/DMSO) ratios were calculated. Mean normalization was used to remove the systemic bias. The normalized ratios for a given protein were tested if their mean was significantly different from “0” using one sample *t*‐test. The FDR multiple testing correction as per Benjamini–Hochberg (B–H) was applied to identify a “top tier” of significant proteins and prevent identification of false positives at an FDR of 5% 9. *p* Values and resulting significant protein lists from the SIDL data and iTRAQ data were updated with PB + PER/DMSO *ln* (natural log) ratios, and uploaded separately to IPA (Ingenuity® Systems, www.ingenuity.com). Briefly, the Ingenuity knowledgebase comprises a repository of proteins/genes that are grouped based on biological interactions and functional relationships derived from published literature and scientific databases. Once a dataset is uploaded to IPA (e.g. in this case, brain proteins demonstrating significant expression changes in response to GW agent exposure), the proteins are mapped onto biological functions and pathways in IPA from which the biological relevance of the response can be inferred as the IPA platform assigns each uploaded protein to particular biofunctions and pathways. IPA uses a right‐tailed Fisher's exact test to calculate a *p*‐value to determine the probability of whether each biofunction and/or disease assigned to that dataset is due to chance alone. Canonical pathways (biological pathways curated by IPA using established scientific databases/literature) most significant to the dataset are determined based on: (i) The ratio of the number of proteins mapping to a pathway divided by the total number of proteins represented in that particular pathway, and (ii) a right‐sided Fisher's exact test is applied to calculate a *p*‐value (*p* < 0.05).

### 2.4 Biochemical analyses investigating mitochondrial dysfunction (MD)

Membrane fractions prepared in the proteomic section were used for all validation studies, as most mitochondrial proteins encompass transmembrane domains, and span the inner and the outer mitochondrial matrix.

#### 2.4.1 Western blotting

Briefly, 25 μg of protein from the membrane‐bound fraction (*n* = 4/ group) was reduced and denatured using Laemmli buffer (Bio‐Rad, Hercules, CA), and resolved on a 4–20% gradient tris‐glycine SDS‐PAGE gel (Bio‐Rad). Proteins were transferred onto PVDF membranes at 90 mA overnight at 4°C, and blocked with 5% w/v powdered milk in TBS. Membranes were probed with mitochondrial antibodies spanning Complex(es) I–V from the oxidative phosphorylation (OXPHOS) chain (Supporting Information Table S1). Positive control was provided with the Total OXPHOS Rodent WB Antibody Cocktail (Abcam, Cambridge, MA). Membranes were then incubated with either anti‐mouse or anti‐rabbit IgG, HRP‐linked secondary antibody (1:1000 dilution; Cell Signaling, Beverly, MA). The Amersham ECL Prime Western Blotting Detection Reagent kit (GE Healthcare) was used for chemiluminescent detection. The signal intensity ratios were quantified by chemiluminescence imaging with Image Lab Software (Bio‐Rad).

##### 2.4.1.1 Statistical analyses of Western blotting experiments

Statistical analyses were performed using Student's *t*‐test with GraphPad Prism 7 (La Jolla, CA). A *p*‐value lower than 0.05 was found to be statistically significant (*p ≤ 0.05; **p ≤ 0.01; ***p ≤ 0.001). Error bars in the figures present the SEM.

### 2.5 Multiplex cytokine assay

The Bio‐Plex multiplex cytokine assay kit (Bio‐Rad) was used to investigate the effects of GW agent exposure on cytokine and chemokine profiles of GW agent treated (*n* = 14) and control (*n* = 14) mouse plasma and brain homogenate samples (plasma samples, 1:4 dilution using Bio‐Plex sample diluent; tissue lysates, 1:2 dilution of lysates at 900 μg/mL of protein in Bio‐Plex sample diluent). The Bio‐Plex Pro™ Mouse Cytokine Th17 Panel A kit (Bio‐Rad) and the Singleplex Interleukin 4 kit (Bio‐Rad and Invitrogen, MD) were used in this study to assess tumor necrosis family‐α (TNF‐α), interferon‐γ, interleukin 6, interleukin 1β (IL‐1β), interleukin 10 (IL‐10), interleukin 17 (IL‐17), and interleukin 4 levels in plasma and brain homogenates, at 5 months postexposure.

## 3 Results

### 3.1 Global proteome profiling of mouse brain homogenate using duplexed iTRAQ and SIDL approaches at 5 months postexposure to GW agents

**Figure 2:**
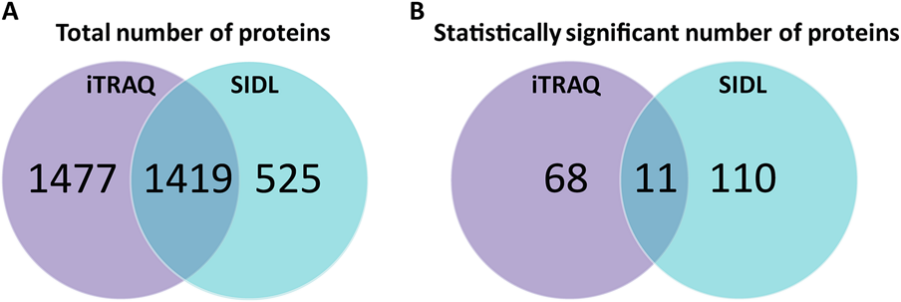
Venn‐diagrams. Venn‐diagram depicting (A) the distinct total number of proteins identified by iTRAQ and SIDL, as well as the overlapping number of proteins. Venn‐diagram illustrating (B) the statistically significant number of proteins identified by iTRAQ and SIDL, unique to each proteomic experiment, as well as the overlapping number of proteins.

**Figure 3:**
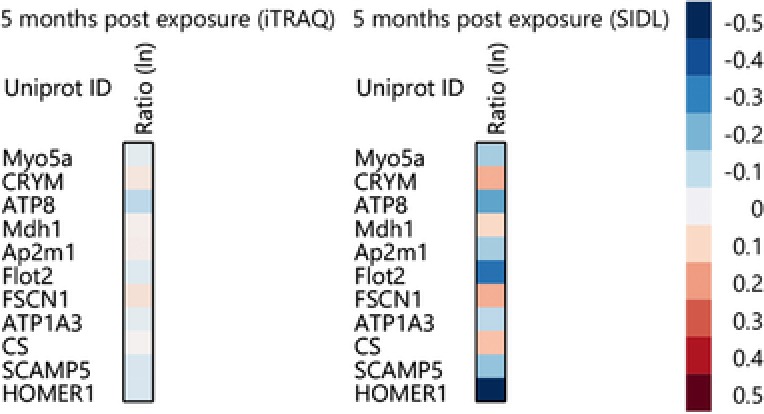
Heatmap of overlapping proteins identified by iTRAQ and SIDL. Heatmap generated from proteomics data (iTRAQ and SIDL) reflecting statistically significant overlapping proteins, and their fold change ratios (ln) in brains of PB + PER exposed as compared to control mice.

The consequences of combined PB + PER exposure in C57BL6/J mice were examined using both iTRAQ and SIDL as complementary survey approaches to identify brain molecular pathways that were impacted by GW agents 5 months postexposure 4. Using iTRAQ, 2896 proteins were quantified between control and exposed mice; of those proteins, 79 proteins were significantly regulated after B–H multiple testing correction. A total of 1944 proteins were identified with SIDL, of which 121 were significantly regulated after B–H multiple testing correction (Fig. 2). Of the 1419 proteins that were identified by both methods, 11 were significantly regulated after B–H multiple testing correction (Fig. 2 and Fig. 3). Those proteins and their functions are described in Table 1. IPA was used to assign biological relevance to the proteomic datasets of significantly modulated proteins, and to identify the highest scoring biological networks. *Neurological Disease, Metabolic Diseases, Skeletal and Muscular Disorders, Immunological and Inflammatory Diseases*, and *Inflammatory Response* were among the top diseases and biofunctions identified by IPA to be significantly associated with GW agent exposure.

**Table 1:** Statistically significant overlapping proteins at 5 months postexposure between iTRAQ and SIDL

Uniprot ID	Description	Ratio (ln) (iTRAQ)	B–H (iTRAQ)	Ratio (ln) (SIDL)	B–H (SIDL)
Ap2m1	AP‐2 complex subunit mu	4.65 × 10^−2^	9.15 × 10^−4^	−1.40 × 10^−1^	2.26 × 10^−3^
ATP1A3	Sodium/potassium‐transporting ATPase subunit alpha‐3	−3.62 × 10^−2^	9.50 × 10^−4^	−1.06 × 10^−1^	1.18 × 10^−3^
ATP8	ATP synthase protein 8	−1.08 × 10^−1^	2.59 × 10^−4^	−2.33 × 10^−1^	2.19 × 10^−3^
CRYM	Ketimine reductase mu‐crystallin	6.68 × 10^−2^	1.29 × 10^−3^	1.72 × 10^−1^	9.00 × 10^−4^
CS	Citrate synthase, mitochondrial	3.59 × 10^−2^	1.33 × 10^−3^	1.43 × 10^−1^	8.23 × 10^−4^
Flot2	Flotillin‐2	−4.91 × 10^−2^	4.83 × 10^−4^	−3.37 × 10^−1^	1.88 × 10^−3^
FSCN1	Fascin	8.36 × 10^−2^	8.63 × 10^−4^	1.71 × 10^−1^	1.52 × 10^−3^
HOMER1	Homer protein homolog 1	−5.77 × 10^−2^	1.16 × 10^−3^	−4.88 × 10^−1^	2.11 × 10^−3^
Mdh1	Malate dehydrogenase, cytoplasmic	4.19 × 10^−2^	2.24 × 10^−4^	1.02 × 10^−1^	2.73 × 10^−3^
Myo5a	Unconventional myosin‐Va	−3.81 × 10^−2^	3.11 × 10^−4^	−1.40 × 10^−1^	1.41 × 10^−3^
SCAMP5	Secretory carrier‐associated membrane protein 5	−6.08 × 10^−2^	1.24 × 10^−3^	−1.68 × 10^−1^	3.06 × 10^−3^

Lists of the overlapping statistically significant proteins after Benjamini–Hochberg (B–H) multiple testing corrections along with their *p* values, performed at 5 months postexposure to GW agents using iTRAQ and SIDL.

**Figure 4:**
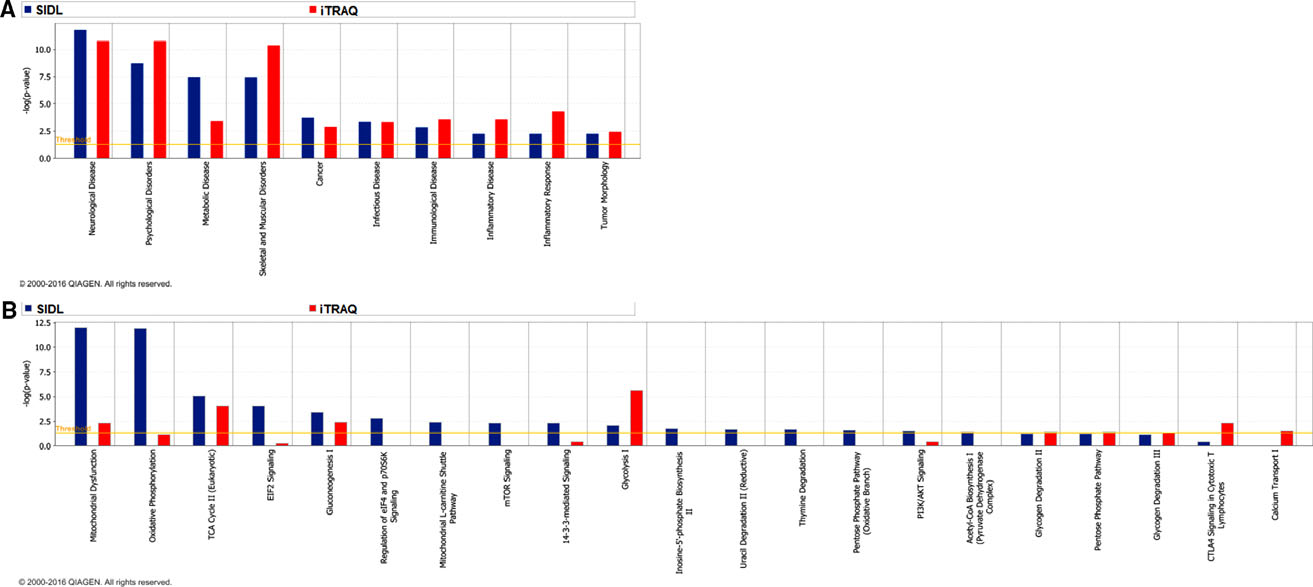
IPA‐based diseases and biofunctions, and canonical pathways. IPA platform analysis mapped top diseases and biofunctions, and top canonical pathways that were associated with CNS changes at 5 months postexposure to GW agent exposure using SIDL and iTRAQ. (A) Top diseases and biofunctions include *Neurological Disease, Psychological Disorders, Metabolic Disease and Skeletal* and *Muscular Disorders*, among others. (B) Top canonical pathways associated with energy production such as Mitochondrial Dysfunction, OXPHOS, and the TCA cycle, as well as immune and inflammatory dysregulation such as mTOR signaling and PI3K/AKT signaling were among the top‐ranking pathways that were affected by GW agent exposure. TCA, tricarboxylic acid.

The Top 5 scoring IPA canonical pathways shown to be affected postexposure that were identified by both proteomic methods were as follows: *MD*, OXPHOS, the *tricarboxylic acid cycle*, *EIF2 Signaling*, and *Gluconeogenesis I* (Fig. 4). Some of these differentially regulated proteins were succinate dehydrogenase complex, subunit A, cytochrome c oxidase subunit 6C (COX6C), NADH dehydrogenase (ubiquinone) Fe‐S protein 4 (NDUFS4), and ATP synthase, H+ transporting, mitochondrial Fo complex, subunit F6 (ATP5J). The SIDL analysis revealed that mitochondrial proteins belonging to Complexes I, II, IV, and V of the OXPHOS network were downregulated in PB + PER mice. Similarly, iTRAQ analysis revealed that Complex V proteins were downregulated in PB + PER mice at 5 months postexposure. While the directional changes for these proteins were consistent between iTRAQ and SIDL datasets, the relative fold changes were not consistent, most likely due to reporter ion signal compression when performing iTRAQ‐based quantification using MS^2^ spectra only. Furthermore, proteins such as inositol 5'‐phosphatase/synaptojanin 1 and heat shock protein 90 kDa alpha (cytosolic), class A member 1 belonging to other canonical pathways such as mechanistic target of rapamycin (mTOR) signaling and phosphoinositide 3‐kinase (PI3K)/AKT signaling which have been recognized as critically important in coordinating defense mechanisms in the innate immune system and in regulating metabolism, cell growth, and survival were also shown to be significantly regulated.

### 3.2 Validation of canonical pathway data implicating MD

#### 3.2.1 Western blotting

**Figure 5:**
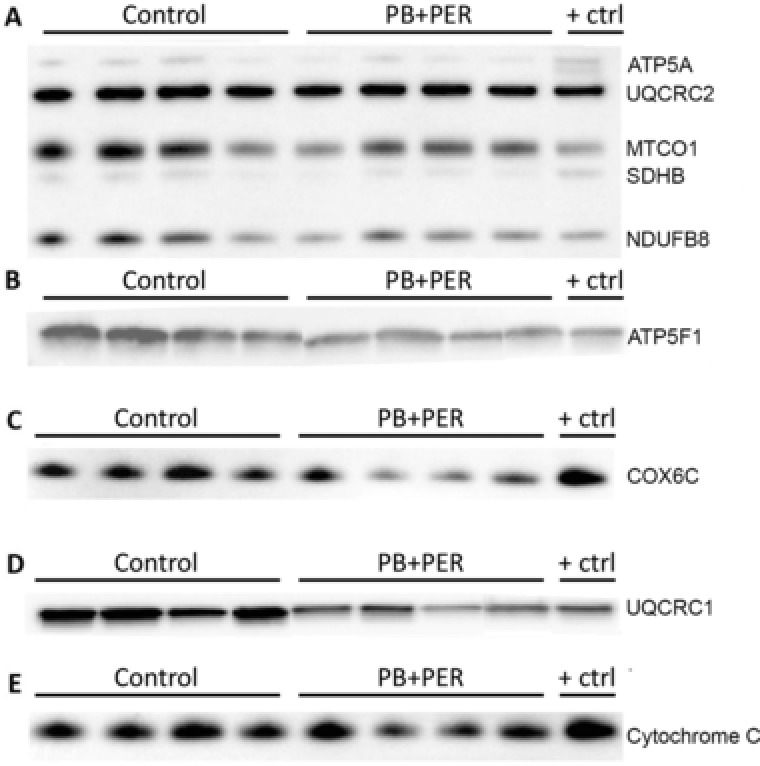
Western blotting analyses using antibodies against mitochondrial proteins. Membrane brain homogenates from control and PB + PER exposed mice were probed with antibodies against mitochondrial proteins. (A) Anti‐OXPHOS cocktail antibody was used to probe membranes using five major proteins (Complex V, ATP5A, 55 kD, Complex III, UQCR2, 48 kD, Complex IV, MTCO1, 40 kD, Complex II, SDHB, 30 kD, and Complex I, NDUFB8, 20 kD) revealed decreased expression in several key proteins such as NDUFB8 (Complex I; *t*‐test = 3.672, DF = 5, *p* = 0.014), SDHB (Complex II; *t*‐test = 3.672, DF = 5, *p* = 0.014), and MTCO1 (Complex IV; *t*‐test = 2.856, DF = 5, *p* = 0.036) in the brains of PB + PER mice as compared to controls. (B) Membranes were probed with anti‐ATP5F1 antibody (Complex V; 29 kD), no statistically significant differences were observed between the two groups. (C) Membranes were probed with an anti‐COX6C antibody (Complex IV, 9 kD), a significant reduction in COX6C expression was revealed in exposed mice as compared to controls (*t* = 3.472, DF = 4, *p* = 0.026). (D) Probing with an anti‐UQCRC1 antibody (Complex III, 35 kD) using brain homogenates revealed a significant reduction in exposed mice as compared to controls (*t*‐test = 3.947, DF = 6, *p* = 0.008). (E) Probing with anti‐cytochrome c antibody (15 kD) revealed no statistically significant differences between the two groups. All protein expression values were normalized to UQCRC2, as a loading control. ATP5A, ATP Synthase, H+ Transporting, Mitochondrial F1 Complex, Alpha Subunit 1; ATPF1, ATP Synthase Mitochondrial F1 Complex Assembly Factor 1; COX6C, Cytochrome C Oxidase Subunit 6C MTCO1, mitochondrially encoded cytochrome c oxidase I; SDHB, succinate dehydrogenase complex, subunit B; UQCRC1, Ubiquinol‐Cytochrome C Reductase Core Protein I; UQCRC2, Ubiquinol‐Cytochrome C Reductase Core Protein II.

**Figure 6:**
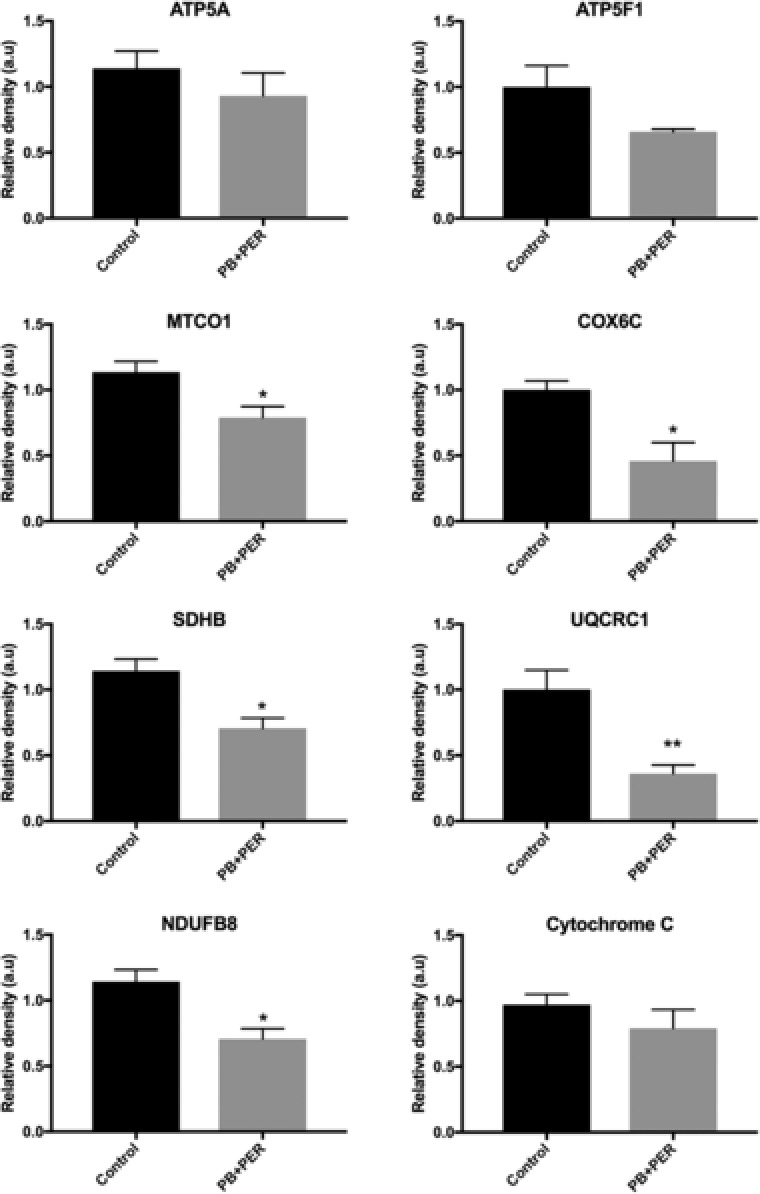
Quantifications of Western blotting data. The histograms represent the average ratio of each representative antibody against UQCRC2 total protein used as a loading control for control and in PB + PER exposed mice. UQCRC2, Ubiquinol‐Cytochrome C Reductase Core Protein II.

Membrane fractions from brain homogenates from control and PB + PER exposed mice were used to validate the alterations in mitochondrial function that were identified by both proteomic methods, at the 5‐month time point. A cocktail of antibodies for the following five major proteins (ATP5A, UQCR2, mitochondrially encoded cytochrome c oxidase I, succinate dehydrogenase complex, subunit B, and NDUFB8) from the OXPHOS complex was used to assay the membrane fraction via Western blot analysis. The immunoblot panel (Fig. 5) shows quite clearly that the Ubiquinol Cytochrome C Reductase Core Protein II signal is unchanged relative to the rest of the proteins; Ubiquinol Cytochrome C Reductase Core Protein II has been listed as a housekeeping gene in some systems 10, 11. We acknowledge that like with most other housekeepers, there are data which show differential expression of this protein in certain experimental paradigms, but this is clearly not the case in our data. Therefore, for the purposes of our studies we have used this protein as a surrogate control. From this, we observed decreased expression consistent with the iTRAQ/SIDL analyses for several key proteins such as NDUFB8 (Complex I; *t*‐test = 3.672, DF = 5, *p* = 0.014), succinate dehydrogenase complex, subunit B (Complex II; *t*‐test = 3.672, DF = 5, *p* = 0.014), mitochondrially encoded cytochrome c oxidase I (Complex IV; *t*‐test = 2.856, DF = 5, *p* = 0.036) in the brains of PB + PER mice as compared to controls (Figs. 5 and 6). Proteomic data also indicated downregulation of Complexes IV and V in PB + PER mice, and so Western blotting with anti‐COX6C and anti‐ATP5F1 antibodies was performed. A significant reduction in COX6C expression was revealed in exposed mice as compared to controls (*t* = 3.472, DF = 4, *p* = 0.026; Figs. 5 and 6). Similarly, a trend toward reduction in anti‐ATP5F1 antibody signal from brain homogenates was observed in the brains of exposed mice relative to controls (*t* = 1.766, DF = 5, *p* = 0.138; Figs. 5 and 6).

In addition, we investigated whether or not other mitochondrial proteins such as Ubiquinol Cytochrome C Reductase Core Protein I (Complex III) were affected, as proteins from Complexes I, II, and III showed a trend toward reduced expression in PB + PER exposed mice when probed with the OXPHOS antibody cocktail. Probing brain homogenates using an anti‐Ubiquinol Cytochrome C Reductase Core Protein I antibody revealed a significant reduction in expression in exposed mice relative to controls (*t*‐test = 3.947, DF = 6, *p* = 0.008; Figs. 5 and 6). Interrogating samples with an anti‐Cytochrome C antibody did not reveal differences in relative protein expression between brains of exposed mice and controls (*t*‐test = 1.104, DF = 6, *p* = 0.312; Figs. 5 and 6).

### 3.3 Multiplex cytokine assay (Bio‐Plex)

**Figure 7:**
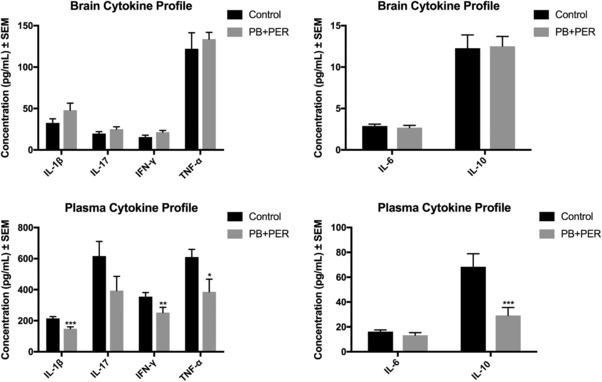
Cytokine profiling. Cytokine profile in brain homogenates and plasma of control and PB + PER exposed mice (*n* = 14/group; *N* =28), using a Bioplex multiplex system for simultaneous detection of Th17 cytokines. (A and B) Concentrations of cytokines in brain homogenates of control and PB + PER exposed mice, no differences were observed between exposed and control mice. (C and D) Concentrations of cytokines in plasma samples of mice, where IFN‐γ (*t*‐test = −3.0, DF = 24, *p* = 0.006), TNF‐α (*t*‐test = −2.4, DF = 24, *p* = 0.02), IL‐10 (*t*‐test = −3.79, DF = 24, *p* = 0.0009), and IL‐1β (*t*‐test = −3.85, DF = 24, *p* = 0.0008) expression was reduced in PB + PER mice as compared to controls. A *p*‐value lower than 0.05 was found to be statistically significant (*p ≤ 0.05; **p ≤ 0.01; ***p ≤ 0.001). Error bars in the figures present the SEM.

Cytokine and chemokine production was analyzed in plasma and brain samples from GW agent exposed mice (*n* = 14) and their controls (*n* = 14), at the 5‐month time point. In plasma, cytokines such as IFN‐γ (*t*‐test = −3.0, DF = 24, *p* = 0.006), TNF‐α (*t*‐test = −2.4, DF = 24, *p* = 0.02), IL‐10 (*t*‐test = −3.79, DF = 24, *p* = 0.0009), and IL‐1β (*t*‐test = −3.85, DF = 24, *p* = 0.0008) were all decreased in the GW agent exposed animals compared to controls (Fig. 7). Plasma interleukin 6 levels were similar in the exposed and control animals (*t*‐test = −1.20, DF = 24, *p* = 0.24), as were the IL‐17 levels (*t*‐test = −1.67, DF = 24, *p* = 0.11). Results from the interleukin 4 singleplex assay (Bio‐Rad and Invitrogen) fell below the LOD when analyzing plasma and brain homogenate samples. This cytokine panel was also used to assay brain homogenates, but no exposure‐dependent differences in cytokine or chemokine levels were observed between control and PB + PER exposed mice (Fig. 7).

## 4 Discussion

### 4.1 Application of complementary proteomic approaches

Two orthogonal but complementary proteomic approaches—SIDL, which utilizes AUC data from full‐scan MS spectra, and iTRAQ, which allows for multiplex quantification from a single MS^2^ spectrum—were utilized to better understand the biochemical pathways that are affected by GW agent exposure. The decision to use these methods in this study was based on several attributes of each. SIDL and iTRAQ are similar in that they are both “bottom‐up” approaches that rely on in vitro isotopic labeling of digested protein samples for multiplexed analysis. Additionally, both typically enable robust quantification of as many as several thousand proteins within a few hours of instrument run time. They are fundamentally different, however, on the means by which samples can be multiplexed, along with the instrumentation and computational parameters required to handle the quantitative aspects of each approach 12, 13, 14. Given such dissimilarities, it can even be reasoned that SIDL and iTRAQ can serve as a de facto validation of one another.

For both approaches, we observed a consistent directional shift in the relative fold changes of proteins following GW agent administration for those portions of their datasets which overlapped, indicating that either approach is sufficient to identify differential changes in complex systems. Though directionally consistent, the calculated fold changes differed considerably (Fig. 3) between the two. Several publications in recent years have documented the effects of signal compression in the reporter ion region of MS^2^ spectra in iTRAQ (or TMT) experiments that arise when isobars, or near isobars (as much as ±0.6 *m/z*, as in the case of this work) are co‐isolated and co‐fragmented 15, 16, 17, 18, 19. In these cases, the sequence‐informative ions present in chimeric MS^2^ spectra can be correctly assigned to their corresponding precursors using conventional search algorithms, however accurate quantitative assignment of reporter ions as a function of precursor abundance in these spectra remains more challenging in fragmentation schemes that are enabled for MS/MS spectra only.

By contrast, fold changes in our SIDL datasets were often more pronounced—a likely consequence of averaging signal intensities of high resolution full‐scan spectra over their entire elution profile. As the majority of the *m/z* values for precursor ions from the full‐scan MS spectra are less than 2 ppm mass error, strict integration parameters can be employed during data analysis to further increase the quantitative stringency of the assay. While this represents a clear improvement in quantitative accuracy over iTRAQ, the assay is limited in terms of throughput, as most SIDL studies are performed in duplex. Multiplexed SIDL studies have been carried out 6, 20, 21, but those approaches are hindered by isotope overlap effects (which increase as a function of increasing precursor mass), as the minimum mass difference is 4 Da for SIDL groupings of three and four, while the minimum mass difference for duplex is 8 Da. Therefore, the use of these combined approaches allowed for greater coverage of proteins than was possible by either one alone, and revealed both distinct and overlapping datasets. In addition, our comparative analysis of the two different datasets allowed for a broader, deeper, and better understanding of the biochemical pathways that were affected by GW agent exposure at the 5‐month time point than would have been possible with one single method.

### 4.2 Proteomic profiling revealed immune/inflammatory dysregulation postexposure to PB + PER

Proteomic analyses by SIDL and iTRAQ revealed immune/inflammatory dysregulation. Both methods demonstrated that Glial fibrillary acidic protein (GFAP) a well‐known marker for reactive astrogliosis and an indicator of neurologic damage, was increased in the brains of PB + PER mice. These data are also consistent with our previously published neuropathological data which revealed an increase in GFAP staining in the brains of GW agent exposed mice at this time point 4. In addition, these findings of immune and inflammatory dysregulation are not only bolstered by the data from the IPA‐based canonical pathways but also by the IPA‐based diseases and biofunctions feature, which revealed *Immunological Diseases, Inflammatory Diseases and Inflammatory Responses* as the top scoring biofunctions which were perturbed, after *Neurological Diseases*, and *Metabolic Disorders*. IPA canonical pathways such as mTOR signaling and PI3K/AKT signaling were affected in the brains of PB + PER mice. Recent data indicate that these pathways may also play a role in coordinating defense mechanisms in the innate immune system 22. PI3K and mTOR have been shown to dampen immune cell activation by upregulating the key anti‐inflammatory cytokine IL‐10 and inhibiting proinflammatory cytokines 22. Since the severity of the dysfunction increased with time postexposure, aberrant immune and inflammatory responses postexposure to GW agents were further evaluated by targeted cytokine profiling using brain and plasma samples.

### 4.3 Peripheral and brain cytokine profiling in PB + PER exposed mice 5 months postexposure

Cytokine profiling revealed a mixed immune profile in the plasma. Proinflammatory cytokines, such as IFN‐γ and TNF‐α, as well as IL‐1β were all decreased in the GW agent exposed mice as compared to controls. In addition, the pleiotropic cytokine, IL‐10 was decreased in the plasma of exposed mice while no differences were detected in IL‐17 expression between exposed and control mice. Perturbations in immune profiles of GWI patients have been extensively studied. However, there is no consensus on whether the cytokine profiles induced by GW agents are primarily pro or anti‐inflammatory. The original Rook and Zumla hypothesis states that the immune system of GWI subjects is biased toward a Th2‐cytokine pattern, and that treatment may be possible with regimens that induce a systemic Th1 bias 23. However, several other clinical studies report either a Th1 bias 24, mixed Th1/Th2 25, or Th17 26, 27 profile.

In the brains of exposed mice, we also identified a marginal elevation of IFN‐γ and IL‐1β. In accordance with these data, major depressive episodes are a common comorbid feature reported in GW veterans and are associated with an upregulation of a variety of pro‐ and anti‐inflammatory cytokines including those which have previously been identified as correlates of GWI severity 28, 29, 30, 31, 32. In summary, these findings underscore the heterogeneity in the immune and inflammatory dysregulation in ill GW subjects and the challenges associated with modulating these responses in conjunction with a mixed immune profile.

### 4.4 Proteomic profiling revealed MD postexposure to PB + PER

Recent clinical evidence has shown that MD may be a persistent feature of GWI, indicating that exposure to GW agents may impair mitochondrial function and compromise mitochondrial integrity 33, 34, 35. Mitochondria are the major source of intracellular ROS, overproduction of which damages proteins, lipids, and nucleic acids, and are thought to contribute to the pathogenesis of many illnesses and diseases that affect the CNS. Proteomic profiling using brain homogenates of mice postexposure to PB + PER revealed that canonical pathways such as the tricarboxylic acid cycle, MD, and glycolysis were differentially regulated after exposure.

### 4.5 Validation of MD

Proteomic analyses demonstrated that proteins from Complexes IV and V were downregulated in PB + PER mice. Cytochrome c oxidase (COX) is part of the Complex IV of the mitochondrial respiratory chain and is responsible for the transfer of an electron from reduced cytochrome c to oxygen. We identified significant reduction of the COX subunit (COX6C) in PB + PER exposed mice as compared to controls. This is in agreement with several other studies which have described a decrease in COX activity after exposure to pesticides and AChE inhibitors 36, 37. In addition, several subunits of Complex V were also shown to be differentially expressed in the brains of exposed mice by both SIDL and iTRAQ approaches. Western blotting revealed a trend toward reduced expression of the Complex V protein, ATP5F1 in the brains of exposed mice compared to controls. Disruption of the Complex V of mitochondria has been shown to have a large effect on the respiratory rate capacity, reducing it by as much as 50% and effectively impairing the synthesis of ATP 38. Thus, the decreased ATP synthase activity witnessed in our model could be indicative of a mild but chronic bioenergetic disruption in the brains of GW agent exposed mice.

### 4.6 Synergistic relationship between MD and inflammation as a consequence of GW agent exposure

Damage to the mitochondrial respiratory chain has been linked to widespread activation of neuroinflammatory processes 39, suggesting that mitochondrial damage may propagate neuroinflammation. Specifically, inhibition of Complex I, the first enzyme of the mitochondrial respiratory chain, has been shown to induce inflammation in the CNS 39, 40. During neuroinflammatory processes, free radicals such as ROS and reactive nitrogen species destroy pathogens within the local environment. However, these molecules can also become oxidized, and in turn cause damage to proteins, nucleic acids, polysaccharides, and lipids, leading to mitochondrial damage 39, 41, 42. Pathological nitrosative stress by the ROS and reactive nitrogen species, resulting in inhibition of COX and further mitochondrial damage, has been implicated in the pathogenesis of many neurodegenerative and neuroinflammatory disorders 43, 44, 45, 46, 47. While an acute neuroinflammatory response may initially be beneficial to the CNS 48, 49, chronic and persistent neuroinflammation may propagate a self‐perpetuating deleterious response which can persist long after the initial event 32, 39. It is hypothesized that, irrespective of the primary pathogenic mechanism (inflammatory or mitochondrial), the multifaceted and synergistic relationship between neuroinflammatory processes and MD may result in feed‐forward loop which can culminate in neurodegeneration. Therefore, the alterations in mitochondrial proteins and immune and inflammatory responses, as well as the interplay between both processes, may be of great importance in understanding the pathobiology of GWI and certainly warrants further study.

The authors declare that the research was conducted in the absence of any commercial or financial relationships that could be construed as a potential conflict of interest.

## Supporting information

Supplementary Table 1: Mitochondrial antibodies spanning Complex(es) I–V from the OXPHOS chain including dilutions used, manufactures and catalogue numbers.
