# Supplementary figures and images for: Complementary proteomic approaches reveal mitochondrial dysfunction, immune and inflammatory dysregulation in a mouse model of Gulf War Illness
Source: Proteomics Clin Appl. 2017 May 12;11(9-10):1600190. doi: 10.1002/prca.201600190 (PMC5637931; doi:10.1002/prca.201600190)

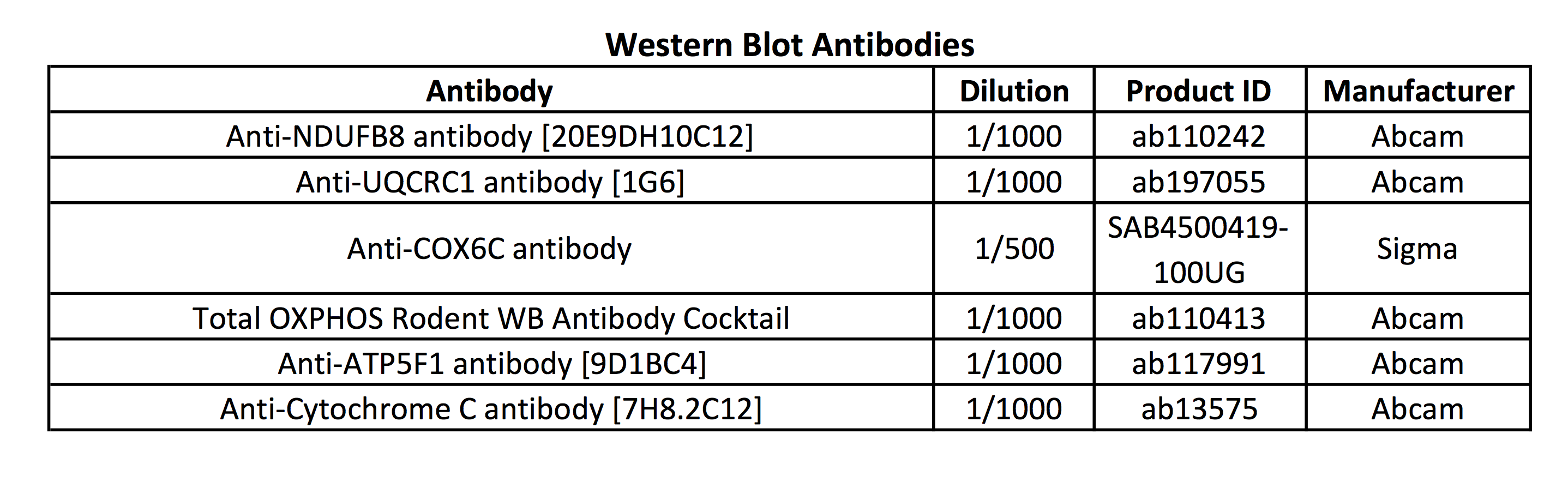

Supplement: Supplementary file 1 — Supplementary Table 1: Mitochondrial antibodies spanning Complex(es) I–V from the OXPHOS chain including dilutions used, manufactures and catalogue numbers. [file PRCA-11-na-s001.tiff]
